# Antimicrobial utilization and antimicrobial resistance in patients with haematological malignancies in Japan: a multi-centre cross-sectional study

**DOI:** 10.1186/s12941-020-00348-0

**Published:** 2020-02-17

**Authors:** Wataru Mimura, Haruhisa Fukuda, Manabu Akazawa

**Affiliations:** 1 Department of Public Health and Epidemiology, Meiji Pharmaceutical University, Kiyose, Tokyo Japan; 2 Department of Health Care Administration and Management, Kyushu University Graduate School of Medical Sciences, Fukuoka, Japan

**Keywords:** Antibiotic resistance, Haematological malignancies, Antimicrobial utilization, Japan, Antimicrobial use density

## Abstract

**Background:**

Infection is a major complication for patients with haematological malignancies. It is important to better understand the use of antimicrobial agents and antibiotic resistance for appropriate treatment and prevention of drug resistance. However, very few multi-centre analyses have focused on the use of antimicrobial agents and antibiotic resistance have been carried out in Japan. This study aimed to describe the characteristics of the use of antimicrobial agents and antibiotic resistance in patients with haematological malignancies.

**Methods:**

We conducted a cross-sectional study using administrative claims data and antimicrobial susceptibility data in Japan. We included patients diagnosed with haematological malignancies, who were hospitalized in a haematology ward between 1 April 2015 and 30 September 2017 in 37 hospitals. Descriptive statistics were used to summarize patient characteristics, antimicrobial utilization, bacterial infections, and antibiotic resistance.

**Results:**

In total, 8064 patients were included. Non-Hodgkin lymphoma (50.0%) was the most common malignancy. The broad-spectrum antibiotics displayed a following antimicrobial use density (AUD): cefepime (156.7), carbapenems (104.8), and piperacillin/tazobactam (28.4). In particular, patients with lymphoid leukaemia, myeloid leukaemia, or myelodysplastic syndromes presented a higher AUD than those with Hodgkin lymphoma, non-Hodgkin lymphoma, or multiple myeloma. The most frequent bacterial species in our study cohort was *Escherichia coli* (9.4%), and this trend was also observed in blood specimens. Fluoroquinolone-resistant *E. coli* (3.6%) was the most frequently observed antibiotic-resistant strain, while other antibiotic-resistant strains were rare.

**Conclusions:**

Broad-spectrum antibiotics were common in patients with haematological malignancies in Japan; however, antibiotic-resistant bacteria including carbapenem-resistant or multidrug-resistant bacteria were infrequent. Our results provide nationwide, cross-sectional insight into the use of antimicrobial agents, prevalence of bacteria, and antibiotic resistance, demonstrating differences in antimicrobial utilization among different haematological diseases.

## Background

Antimicrobial resistance (AMR) is a worldwide concern. In accordance with the Global Action Plan on Antimicrobial Resistance from the World Health Organization, a National Action Plan on Antimicrobial Resistance was adopted in 2016 in Japan [1]. AMR is associated with a high mortality rate and increases both healthcare resource utilization and medical costs [2]. The incidence of gram-negative bacterial infections has increased among cancer patients [3–5]. Furthermore, some studies have reported the emergence of broad-spectrum antibiotic-resistant bacteria, including multidrug-resistant *Pseudomonas aeruginosa* (MDRP) and *Acinetobacter* spp. (MDRA), carbapenem-resistant Enterobacteriaceae (CRE) and *P. aeruginosa* (CRP), third-generation cephalosporin-resistant *Escherichia coli* and *Klebsiella pneumonia*, and methicillin-resistant *Staphylococcus aureus* (MRSA) [6–9].

These antibiotic-resistant strains have become a significant threat to cancer patients [9–12]. In particular, infections are a major complication for patients with haematological malignancies because they exhibit certain risk factors for infection, such as malignancies itself, chemotherapy, neutropenia, and hematopoietic stem cell transplantation. Therefore, broad-spectrum antibiotics and antimicrobial prophylaxis for patients with a high risk of febrile neutropenia should be administered in accordance with specific guidelines (e.g. advanced age, patients with lymphoma, multiple myeloma, chronic lymphocytic leukaemia, acute leukaemia, or neutropenia) [13, 14]. The characteristics of AMR and antimicrobial consumption differ among countries, regions, and patient groups [15–18]. In Japan, ceftriaxone has been the major antibiotic administered parenterally, followed by cefazolin, ampicillin/sulbactam, meropenem, piperacillin/tazobactam, etc. while clarithromycin has been the major antibiotic administered orally, followed by cefcapene, levofloxacin, cefditren, amoxicillin, etc. [19, 20]. And MRSA has been the most prevalent AMR bacteria since 2008 [17].

However, a previous study on the use of antibiotics in Japan reported an increase in the antimicrobial use density (AUD) in haematology wards and low antimicrobial susceptibility in comparison with all other hospital departments [21]. Other studies have reported that an increase in the use of antibiotics increases the frequency of occurrence of AMR [22, 23].

To prevent and minimize AMR and to recommend appropriate antibiotics, it is first important to understand the use of antibiotics and infections in patients with haematological malignancies. However, few multi-centre studies on this issue have been conducted in Japan, as most studies have been conducted at a single centre. Therefore, this study aimed to describe the characteristics of antimicrobial utilization and antimicrobial resistance for patients with haematological malignancies.

## Methods

### Study design and setting

We conducted a cross-sectional study using administrative claims data, discharge summary data, and antimicrobial susceptibility test data among 37 acute-care hospitals in Japan. In total, 145 hospitals agreed to provide and analyse the data, and 37 hospitals were confirmed to have an existing haematology ward from inpatient data. These 37 hospitals had capacities of < 200 beds (1 hospital), 200–399 beds (12 hospitals), and > 400 beds (24 hospitals). The administrative claims data and discharge summary data were based on the diagnostic procedure combination/per-diem payment system (DPC/PDPS) [24]. The DPC data included patient information regarding baseline characteristics (e.g. age, sex, disease, and the International Classification of Disease 10th revision [ICD-10] code) and medical procedures (e.g. prescription, surgery, examination, procedure, their codes, and cost) between April 1, 2015 and September 30, 2017. To analyse bacterial and antimicrobial susceptibility, we also used the results of susceptibility tests based on the Japan Nosocomial Infections Surveillance (JANIS) programme conducted by the Ministry of Health, Labour and Welfare on the prevalence of antibiotic-resistant bacteria, using data from multiple hospitals [17, 25]. The JANIS data provided information regarding patient demographics, specimen reception date, specimen sources, types of bacteria, and susceptibility tests results. The data encompassed both outpatients’ and inpatients’ records.

Patients admitted to a haematology ward after April 1, 2015 and diagnosed with at least one haematological malignancy were included herein. Patients with haematological malignancies were classified as ICD-10 of C81 (Hodgkin lymphoma), C82–C85 and C96 (Non-Hodgkin lymphoma), C90 (multiple myeloma), C91 (lymphoid leukaemia), C92-C94 (myeloid leukaemia), or D46 (myelodysplastic syndromes). Among the patients with several haematological malignancies, we selected the diagnosis most closely associated with treatment. When patients were admitted and diagnosed with a haematological malignancy in a haematology ward, we defined the date of admission as the date of indexing. Patients were followed-up from the date of indexing to the date of last discharge; therefore, all patients hospitalized several times were pooled together. Patients without records of hospitalization, procedures, drugs, or surgery were excluded, although such patients were diagnosed with a haematological malignancy in a haematology ward.

To analyse administrative and antibiotic susceptibility data, we used a third-step deterministic linkage process to link DPC and JANIS data. Each linkage step was conducted for data from each hospital. First, we matched two databases on the basis of individual patients, using information regarding the year of birth, month of birth, sex, specimen reception date, and specimen sources. Patients with numerous records of the specimen reception date had higher chances of linkage than those with one record. Therefore, if patients in the DPC database simultaneously linked with multiple patients in the JANIS database, we considered the most frequently observed linkage pairs as matched patient pairs. In the second step, we selected patients who could not be matched in the first step and then re-linked those patients with the year of birth, month of birth, sex, specimen reception date ± 1 day, and specimen sources. In third step, we included two more variables of departments and the date of receipt of specimens from inpatients or outpatients. In addition, we manually reviewed multiple linked patient pairs in third step to determine which pairs were better matched in accordance with the department and periods of hospitalization.

### Variables and outcomes definitions

Comorbidity was assessed in accordance with the Charlson comorbidity index (CCI) [26]. The follow-up period was defined as the duration from the index date to the date of last discharge that we could confirm. Febrile neutropenia was identified on the basis of the disease. Granulocyte-colony stimulating factor (G-CSF) administration was defined as the administration of filgrastim, pegfilgrastim, lenograstim, or nartograstim. We accumulated data on chemotherapy conducted during hospitalization. Central venous catheters, urinary catheters, isolation rooms, and hematopoietic stem cell transplantation (HSCT) were defined by their codes (Additional file 1: Table S1). We assessed antimicrobial utilization (parenteral) and levofloxacin (oral) during hospitalization on the basis of the antimicrobial use density (AUD). To calculate the AUD, we used a defined daily dose (DDD) in accordance with the anatomical therapeutic chemical (ATC) classification system of the WHO (2015 version). In the absence of a DDD in the ATC/DDD system, we used the Japanese DDD defined by the AMR clinical reference centre [27]. We expressed the AUD as DDDs per 1000 patient-days. We calculated the prevalence of bacteria, MDRP, MDRA, CRE, CRP, third-generation cephalosporin-resistant *E. coli*, third-generation cephalosporin-resistant *K. pneumoniae*, fluoroquinolone-resistant *E. coli*, and MRSA during the study period in our cohort. All submitted specimens were assessed to determine the prevalence of bacteria and antimicrobial resistance by the type of specimens (blood, respiratory, urine, and any). These values were calculated from the number of patients with specific bacteria from each specimen as the numerator, and the number of patients in our cohort as the denominator. If the same bacterial species was detected in a patient at different timepoints, we considered only one case. JANIS determined antimicrobial susceptibilities in accordance with the Clinical and Laboratory Standards Institute 2012 guidelines. The definition of antimicrobial-resistant bacteria and the minimum inhibitory concentration values by broth microdilution method based on the JANIS definition are presented in Additional file 2: Table S2 [17].

### Statistical analysis

We used descriptive statistics to summarize patient characteristics (sex, age, underlying disease, CCI, follow-up period, total length of stay [LOS], LOS at one hospitalization, chemotherapy, febrile neutropenia, G-CSF, central venous catheter, urinary catheter, hematopoietic stem cell transplant [allogeneic and autologous], and in-hospital mortality), parental antimicrobial and oral fluoroquinolone utilization stratified by class, period of prevalence of bacteria (from all specimens and blood, respiratory, urine, and stool specimens), and detection of antibiotic-resistant bacteria. We presented continuous variables as median values (quantile range; Q1–Q3) and categorical variables as numbers and percentages (%). All data were analysed using SAS, version 9.4 software (SAS Institute, Inc., Cary, NC, USA).

## Results

The number of patients who discharged from 37 hospitals was 712,335, and we identified 8064 patients who met our inclusion criteria between April 1, 2015 and August 31, 2017. The number of patients at each hospital (median, Q1–Q3) was 204.5 (112–307). Only 2 patients were excluded from this study because of a lack of records. Males constituted 56.8% of the patient population and the median age (Q1–Q3) was 70 (61–78) years (Table 1). Patients aged 75 years or older constituted the majority (36.2%) and those aged 65–74 years constituted 31.1% of the patient population. Approximately half of the patients were diagnosed with non-Hodgkin lymphoma (50.0%), followed by myeloid leukaemia (15.2%), multiple myeloma (14.3%), myelodysplastic syndromes (11.3%), lymphoid leukaemia (6.8%), and Hodgkin lymphoma (2.5%). The follow-up duration (median) was 97 days and the total LOS (median) was 50 days. Chemotherapy was administered to 81.1% of patients and the frequency of patients diagnosed with febrile neutropenia was 13.6%. A central venous catheter (38.1%) was used more frequently than a urinary catheter (26.2%). In total, 2352 (29.2%) of patients were placed in an isolation room. Allogeneic and autologous HSCT was conducted for 5.0% and 3.7% of patients, respectively. In-hospital mortality among all patients was 21.8%, whereas patients with myelodysplastic syndromes displayed 35.1% in-hospital mortality (Additional file 3: Table S3).

**Table 1:** Characteristics of patients with haematological malignancies

Characteristics	Patients (n = 8064)
Sex, n (%)	
Male	4578 (56.8)
Female	3486 (43.2)
Age, years, median (Q1–Q3)	70 (61–78)
≤ 17 years	17 (0.2)
18–64 years	2624 (32.5)
65–74 years	2504 (31.0)
≥ 75 years	2919 (36.2)
Underlying disease, n (%)	
Hodgkin lymphoma	199 (2.5)
non-Hodgkin lymphoma	4028 (50.0)
Multiple myeloma	1153 (14.3)
Lymphoid leukaemia	551 (6.8)
Myeloid leukaemia	1224 (15.2)
Myelodysplastic syndromes	909 (11.3)
CCI, n (%)	
≤ 2	6394 (79.3)
3–5	1392 (17.3)
> 5	278 (3.4)
Follow-up duration, days, median (Q1–Q3)	97 (26–227)
Total LOS, days, median (Q1–Q3)	50 (22–107)
LOS at one hospitalization, days, median (Q1–Q3)	19 (10–30)
Chemotherapy, n (%)	6542 (81.1)
Febrile neutropenia, n (%)	1100 (13.6)
G-CSF, n (%)	4039 (50.1)
Central venous catheter, n (%)	3072 (38.1)
Urinary catheter, n (%)	2109 (26.2)
HSCT, n (%)	
Allogeneic	407 (5.0)
Autologous	301 (3.7)
Isolation room, n (%)	2352 (29.2)
In-hospital mortality, n (%)	1761 (21.8)

*CCI* Charlson comorbidity index, *LOS* length of stay, *G-CSF* Granulocyte-colony stimulating factor, *HSCT* haematopoietic stem cell transportation

**Fig. 1:**
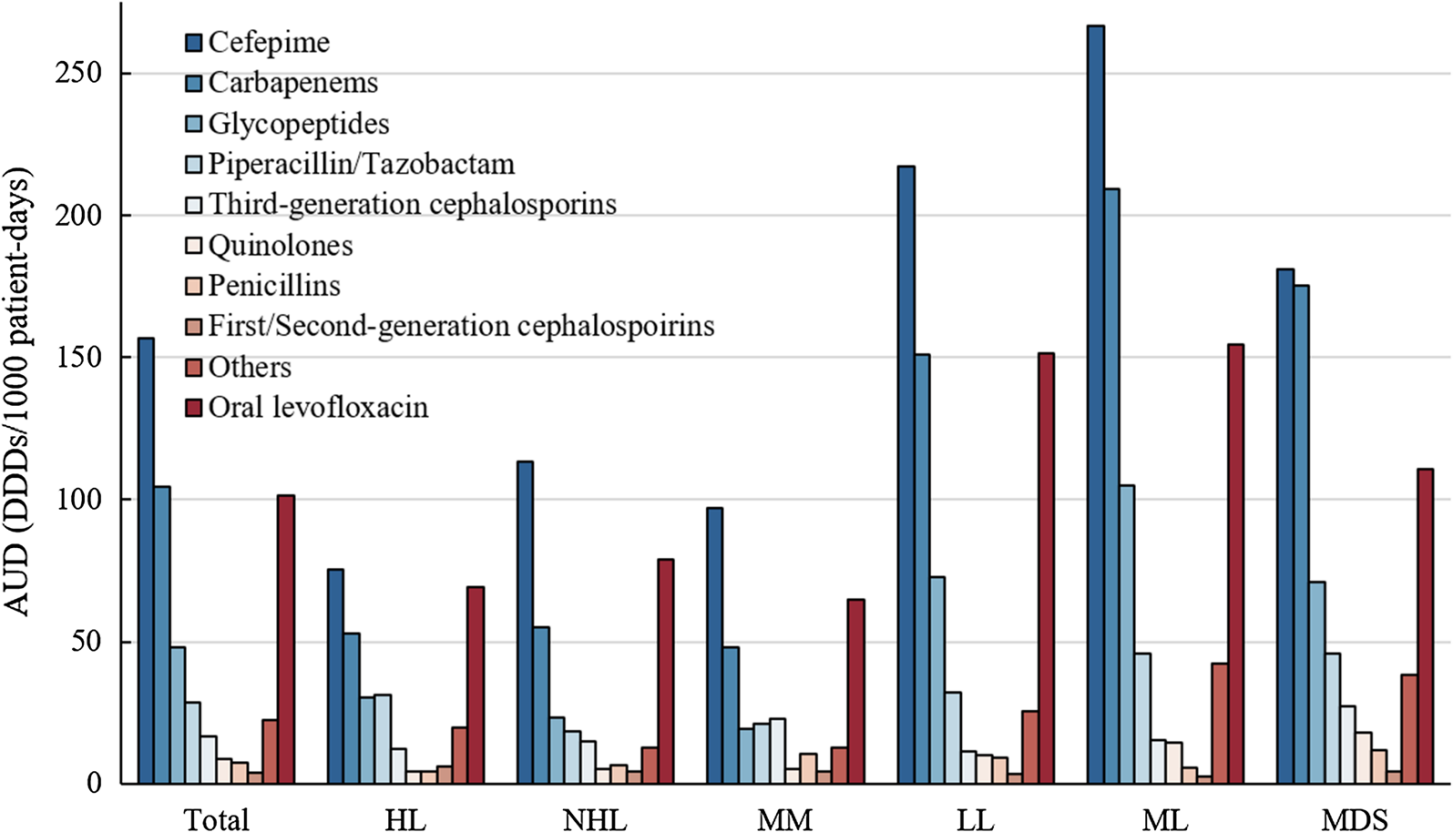
Comparison of antimicrobial use density among underlying diseases. *AUD* antimicrobial use density, *HL* Hodgkin lymphoma, *NHL* non-Hodgkin lymphoma, *MM* multiple myeloma, *LL* lymphoid leukaemia, *ML* myeloid leukaemia, *MDS* myelodysplastic syndromes

Utilization of broad-spectrum antibiotics displayed the following AUD: cefepime (156.7), carbapenems (104.8), and piperacillin/tazobactam (28.4) (Fig. 1). Glycopeptides (48.0) also presented a high AUD, whereas third-generation cephalosporins, quinolones, penicillins, first/second-generation cephalosporins, and others presented AUD values of 16.8, 8.8, 7.5, 4.0, and 22.3, respectively. Furthermore, the AUD of oral levofloxacin was 101.5. Antimicrobial utilization stratified by underlying disease revealed that patients with lymphoid leukaemia, myeloid leukaemia, and myelodysplastic syndromes had a higher AUD than those with other underlying diseases (Additional file 4: Table S4).

The total number of patients submitting any type of specimen was 4963 (61.5%) in the DPC database. In first step of deterministic linkage analysis, 4649 (93.6%) of patients matched DPC data with JANIS data; in the second step, 232 (5.3%) patients matched and in the third step, 8 (0.9%) patients matched. The 8 matched patients’ pairs were determined by manual review. In total, 4889 (98.5%) patients were linked through linkage steps. *Escherichia coli* (9.4%) was the most frequently observed bacterium, followed by *Klebsiella* spp. (5.6%), *P. aeruginosa* (3.5%), *S. aureus* (3.3%), *Enterobacter* spp. (2.7%), *Citrobacter* spp. (2.1%), *Acinetobacter* spp. (1.1%), *Proteus* spp. (0.7%), and *Serratia marcescens* (0.5%) in any specimens. Although only a few blood specimens were positive, the positive results displayed a similar trend (Table 2 and Additional file 5: Table S5). Fluoroquinolone-resistant *E. coli* was the most frequently detected antibiotic-resistant bacterium and was detected in 291 (3.6%) patients. MRSA and third-generation cephalosporin-resistant *E. coli* were detected in 210 (2.6%) and 167 (2.1%) patients, respectively. Overall, the proportion of antibiotic-resistant bacteria was lower and multidrug-resistant bacteria were rarely observed in our patient cohort (Table 3 and Additional file 6: Table S6).

**Table 2:** Detection of bacteria from each specimen

	Patient (n = 8064)			
	Blood (n = 4391)	Respiratory (n = 2000)	Urine (n = 1664)	Any^a^ (n = 4889)
*E. coli*	192 (2.4)	78 (1.0)	224 (2.8)	754 (9.4)
*P. aeruginosa*	73 (0.9)	128 (1.6)	47 (0.6)	282 (3.5)
*Klebsiella* spp.	97 (1.2)	105 (1.3)	84 (1.0)	455 (5.6)
*Enterobacter* spp.	48 (0.6)	74 (0.9)	26 (0.3)	219 (2.7)
*Citrobacter* spp.	9 (0.1)	11 (0.1)	36 (0.4)	167 (2.1)
*Serratia marcescens*	9 (0.1)	21 (0.3)	4 (0.0)	38 (0.5)
*Proteus* spp.	5 (0.1)	3 (0.0)	25 (0.3)	58 (0.7)
*Acinetobacter* spp.	20 (0.2)	62 (0.8)	1 (0.0)	90 (1.1)
*S. aureus*	60 (0.7)	181 (2.2)	18 (0.2)	270 (3.3)

^a^Any include all type of specimens (blood, respiratory, urine, stool, cerebrospinal fluid, and others)

**Table 3:** Detection of antibiotic-resistant bacteria from each specimen

	Patient (n = 8064)			
	Blood (n = 4391)	Respiratory (n = 2000)	Urine (n = 1664)	Any^a^ (n = 4889)
Multidrug-resistant *P. aeruginosa*	0 (0.0)	1 (0.0)	1 (0.0)	2 (0.0)
Multidrug-resistant *Acinetobacter* spp.	0 (0.0)	0 (0.0)	0 (0.0)	0 (0.0)
Carbapenem-resistant Enterobacteriaceae	3 (0.0)	2 (0.0)	4 (0.0)	15 (0.2)
Carbapenem-resistant *P. aeruginosa*	13 (0.2)	23 (0.3)	9 (0.1)	45 (0.6)
Third-generation cephalosporin-resistant *K. pneumoniae*	10 (0.1)	9 (0.1)	15 (0.2)	42 (0.5)
Third-generation cephalosporin-resistant *E. coli*	51 (0.6)	29 (0.4)	61 (0.8)	167 (2.1)
Fluoroquinolone-resistant *E. coli*	104 (1.3)	40 (0.5)	112 (1.4)	291 (3.6)
Methicillin-resistant *S. aureus*	45 (0.6)	141 (1.7)	16 (0.2)	210 (2.6)

^a^Any include all type of specimens (blood, respiratory, urine, stool, cerebrospinal fluid, and others)

## Discussion

This multi-centre cross-sectional study was performed to describe the characteristics of antimicrobial utilization and infections caused by specific bacteria and antibiotic-resistant bacteria and reported high AUD values for cefepime and carbapenems. In particular, patients with lymphoid leukaemia, myeloid leukaemia, and myelodysplastic syndromes reported greater antimicrobial utilization than those with Hodgkin lymphoma, non-Hodgkin lymphoma, and multiple myeloma. More generalizable results were obtained for antimicrobial utilization and antibiotic resistance in comparison with previous studies.

Broad-spectrum antimicrobial agents targeting *P. aeruginosa*, such as cefepime, carbapenems, and piperacillin/tazobactam, are often used among patients with haematological malignancies. The results might imply that those antimicrobial agents were used to cover also gram positive bacteria. Carbapenems and β-lactam/β-lactamase inhibitor combinations have been used more frequently in countries other than Japan [28, 29]. However, compared with previous studies, the use of cefepime was markedly high, presenting as a clear difference. Previous studies regarding the use of antibiotics in Japan reported that combinations of penicillin, including β-lactamase inhibitors were used frequently, followed by third-generation cephalosporins [19, 20, 30]. However, the present results show that more broad-spectrum antibiotics were used for specific populations. These results are similar to those of a previous single-centre study regarding antimicrobial utilization in haematology wards in Japan [21, 31]. This similarity is potentially attributed to the inclusion of most patients in this study, who were at intermediate or high risk of infection because of an underlying disease and chemotherapy [13]. Furthermore, we assessed differences in antimicrobial utilization on the basis of the underlying diseases among haematological malignancies, using multi-centre data. Consequently, the risk of febrile neutropenia including lymphoid leukaemia, myeloid leukaemia, and myelodysplastic syndromes increases, thus resulting in a high AUD [14]. Furthermore, glycopeptides displayed a high AUD. According to this results and JANIS data, MRSA was the most common antibiotic-resistant bacterium in Japan [17, 25], suggesting the possibility that numerous patients have MRSA infections or that anti-MRSA agents might be administered to prevent these infections as empirical therapy. Therefore, further studies are required to investigate associations between the use of anti-MRSA agents and MRSA infections.

MDRA and CRE was rarely detected in this study. A previous study reported the AMR prevalence using data from the JANIS database and infections with multidrug-resistant *P. aeruginosa* (0.07%), multidrug-resistant *Acinetobacter* spp. (0.01%), carbapenem-resistant Enterobacteriaceae (0.36%), carbapenem-resistant *P. aeruginosa* (0.84%), third-generation cephalosporin-resistant *K. pneumonia* (0.32%), third-generation cephalosporin resistant *E. coli* (1.99%), and fluoroquinolone-resistant *E. coli* (3.70%) among patients submitting specimens in 2015 [17, 25]. We could not compare each result directly because the calculation methods were slightly different among studies; however, antibiotic-resistant bacteria in the population of patient submitting specimens were more numerous in the present study population than in previous studies.

Fluoroquinolone-resistant *E. coli* was most frequently detected, though the AUD of quinolones (parenteral) was not markedly greater than that of others. Meanwhile, the AUD of levofloxacin (oral) was 104.0 in the total population. These findings imply that quinolone-resistant bacteria were affected via oral administration of fluoroquinolone as antibacterial prophylaxis (FQ) or community treatment. FQ reduced bloodstream infections and febrile neutropenia in patients with neutropenia; however, some studies reported that FQ increases antibiotic resistance [3, 32]. The Japan Adult Leukaemia Study Group (JALSG) reported that FQ (64.0%) was administered as prophylaxis by clinicians who were members of the JALSG [33]. If fluoroquinolone-resistant bacteria emerge, the effectiveness of FQ and its application should be reconsidered. Furthermore, third-generation cephalosporin-resistant *E. coli* were more frequent than other antibiotic-resistant bacteria. This finding may be related to the many patients who were prescribed oral third-generation cephalosporin compared with those in other countries, in an outpatient setting [19, 20, 30, 34].

Overall, the occurrence AMR was not high probably because of the effectiveness of antibiotics, intervention by infection control teams, use of cleanrooms, and standard precautions. Herein, we simply focused on the use of antibiotics and prevalence of bacterial infections; hence, we did not consider such factors or the chronological order between antimicrobial utilization and emergence of antibiotic-resistant bacteria.

This study had several limitations. First, this study included specific acute-care hospitals that agreed to provide their DPC and JANIS data. Therefore, our results may not represent all patients with haematological malignancies. This may have led to a selection bias among patients and data regarding better-controlled infections in these hospitals rather than in average hospitals could be included. Second, we could not identify contamination, colonization, and source of infection. And we could not also distinguish surveillance culture. Therefore, our data may not reflect clinical infections; however, gram-negative bacteria isolated from blood cultures may be considered true-positive systemic infections in comparison with gram-positive bacteria [35]. Third, we linked DPC data to JANIS data using the deterministic linkage method that we had validated using DPC and JANIS datasets with common identification [36]. Although we obtained a high matching proportion, we could not eliminate the possibility of false-matched cases. However, to reduce false matches, we performed deterministic linkage analysis using 5 variables (hospitals, year of birth, month of birth, sex, specimen reception date, and specimen sources) and manual review. Furthermore, each hospital was used as blocking; hence, there was no probability of mismatching of inter-hospital data. And to avoid overestimation of the prevalence, we calculated it based on patients with haematological malignancies, not patient submitting specimens, because we considered that patients with false-negative linkage were more likely to have few specimens’ submission.

Fourth, we did not distinguish that the use of antimicrobial agents was definitive or empirical therapy and elaborate on how to use of antimicrobial agents, because we focused on the antimicrobial use density and prevalence of AMR. Therefore, we could not have clearly conclusion of appropriate antimicrobial use or the association between antimicrobial use and AMR.

## Conclusion

The present results provide nationwide, cross-sectional insight into the characteristics of antimicrobial utilization, bacterial infections, and antibiotic resistance using multi-centre administrative data and antimicrobial susceptibility data in Japan. Broad-spectrum antimicrobial agents were commonly used, although multidrug-resistant bacteria were not observed. However, fluoroquinolone-resistant *E. coli* and third-generation antibiotic-resistant *E. coli* were frequently observed in comparison with other antibiotic-resistant strains. Moreover, our results show a difference in antimicrobial utilization among underlying diseases. Further studies are required to analyse the risk factors and effects of emerging antimicrobial-resistant bacteria.

## Supplementary information

**Additional file 1: Table S1.** Definitions of variables.

**Additional file 2: Table S2.** Definitions of antibiotic-resistant bacteria.

**Additional file 3: Table S3.** Characteristics of patients with haematological malignancies stratified by the underlying disease.

**Additional file 4: Table S4.** Antimicrobial use among underlying diseases

**Additional file 5: Table S5.** Infections caused by gram-negative bacteria stratified by the underlying disease.

**Additional file 6: Table S6.** Infections caused by antibiotic-resistant bacteria stratified by the underlying disease.

## Data Availability

The datasets generated and/or analysed during the current study are not publicly available owing to restrictions.

## Abbreviations

AUD: Antimicrobial use density
AMR: Antimicrobial resistance
MDRP: Multidrug-resistant *Pseudomonas aeruginosa*
MDRA: Multidrug-resistant *Acinetobacter* spp
CRE: Carbapenem-resistant Enterobacteriaceae
CRE: Carbapenem-resistant *Pseudomonas aeruginosa*
MRSA: Methicillin-resistant *Staphylococcus aureus*
DPC: Diagnostic procedure combination
ICD-10: The International Classification of Disease 10th revision
JANIS: Japan Nosocomial Infections Surveillance
CCI: Charlson comorbidity index
HSCT: Hematopoietic stem cell transplantation
G-CSF: Granulocyte-colony stimulating factor
HL: Hodgkin lymphoma
NHL: Non-Hodgkin lymphoma
MM: Multiple myeloma
LL: Lymphoid leukaemia
ML: Myeloid leukaemia
DDD: Defined daily dose
ATC: Anatomical therapeutic chemical
LOS: Length of stay
JALSG: The Japan Adult Leukaemia Study Group
FQ: Fluoroquinolone prophylaxis
MRSA: Methicillin-resistant *Staphylococcus aureus*

## References

[1] The Government of Japan. National Action Plan on Antimicrobial Resistance (AMR) 2016–2020. https://www.mhlw.go.jp/file/06-Seisakujouhou-10900000-Kenkoukyoku/0000138942.pdf. Accessed 27 July 2019.

[2] Fair RJ, Tor Y (2014). Antibiotics and bacterial resistance in the 21st century. Perspect Medicin Chem..

[3] Cattaneo C, Quaresmini G, Casari S, Capucci MA, Micheletti M, Borlenghi E, Signorini L, Re A, Carosi G, Rossi G (2008). Recent changes in bacterial epidemiology and the emergence of fluoroquinolone-resistant Escherichia coli among patients with haematological malignancies: results of a prospective study on 823 patients at a single institution. J Antimicrob Chemother.

[4] Montassier E, Batard E, Gastinne T, Potel G, de La Cochetiere MF (2013). Recent changes in bacteremia in patients with cancer: a systematic review of epidemiology and antibiotic resistance. Eur J Clin Microbiol Infect Dis.

[5] Chen CY, Tsay W, Tang JL, Tien HF, Chen YC, Chang SC, Hsueh PR (2010). Epidemiology of bloodstream infections in patients with haematological malignancies with and without neutropenia. Epidemiol Infect.

[6] Viale P, Giannella M, Lewis R, Trecarichi EM, Petrosillo N, Tumbarello M (2013). Predictors of mortality in multidrug-resistant *Klebsiella pneumoniae* bloodstream infections. Expert Rev Anti Infect Ther..

[7] Ma J, Li N, Liu Y, Wang C, Liu X, Chen S, Xie X, Gan S, Wang M, Cao W (2017). Antimicrobial resistance patterns, clinical features, and risk factors for septic shock and death of nosocomial E coli bacteremia in adult patients with hematological disease: a monocenter retrospective study in China. Medicine.

[8] Kara O, Zarakolu P, Ascioglu S, Etgul S, Uz B, Buyukasik Y, Akova M (2015). Epidemiology and emerging resistance in bacterial bloodstream infections in patients with hematologic malignancies. Infect Dis..

[9] Trecarichi EM, Tumbarello M (2014). Antimicrobial-resistant gram-negative bacteria in febrile neutropenic patients with cancer: current epidemiology and clinical impact. Curr Opin Infect Dis..

[10] Trecarichi EM, Tumbarello M, Caira M, Candoni A, Cattaneo C, Pastore D, Fanci R, Nosari A, Vianelli N, Busca A (2011). Multidrug resistant Pseudomonas aeruginosa bloodstream infection in adult patients with hematologic malignancies. Haematologica.

[11] Trecarichi EM, Cauda R, Tumbarello M (2012). Detecting risk and predicting patient mortality in patients with extended-spectrum beta-lactamase-producing Enterobacteriaceae bloodstream infections. Future Microbiol..

[12] Bastug A, Kayaaslan B, Kazancioglu S, But A, Aslaner H, Akinci E, Yetkin MA, Kanyilmaz D, Eren SS, Bodur H (2015). Emergence of multidrug resistant isolates and mortality predictors in patients with solid tumors or hematological malignancies. J Infect Dev Ctries..

[13] National Comprehensive Cancer Network. Prevention and Treatment of Cancer-Related Infections (Version 1.2019). https://www.nccn.org/professionals/physician_gls/pdf/infections.pdf. Accessed 27 July 2019.

[14] Taplitz RA, Kennedy EB, Bow EJ, Crews J, Gleason C, Hawley DK, Langston AA, Nastoupil LJ, Rajotte M, Rolston KV (2018). Antimicrobial prophylaxis for adult patients with cancer-related immunosuppression: ASCO and IDSA clinical practice guideline update. J Clin Oncol.

[15] Van Boeckel TP, Gandra S, Ashok A, Caudron Q, Grenfell BT, Levin SA, Laxminarayan R (2014). Global antibiotic consumption 2000 to 2010: an analysis of national pharmaceutical sales data. Lancet Infect Dis..

[16] Versporten A, Zarb P, Caniaux I, Gros MF, Drapier N, Miller M, Jarlier V, Nathwani D, Goossens H, Koraqi A, Hoxha I (2018). Antimicrobial consumption and resistance in adult hospital inpatients in 53 countries: results of an internet-based global point prevalence survey. Lancet Glob Health..

[17] Tsutsui A, Suzuki S (2018). Japan nosocomial infections surveillance (JANIS): a model of sustainable national antimicrobial resistance surveillance based on hospital diagnostic microbiology laboratories. BMC Health Serv Res..

[18] Olesen SW, Barnett ML, MacFadden DR, Brownstein JS, Hernandez-Diaz S, Lipsitch M, Grad YH (2018). The distribution of antibiotic use and its association with antibiotic resistance. Elife.

[19] Tsutsui A, Yahara K, Shibayama K (2018). Trends and patterns of national antimicrobial consumption in Japan from 2004 to 2016. J Infect Chemother..

[20] Yamasaki D, Tanabe M, Muraki Y, Kato G, Ohmagari N, Yagi T (2018). The first report of Japanese antimicrobial use measured by national database based on health insurance claims data (2011-2013): comparison with sales data, and trend analysis stratified by antimicrobial category and age group. Infection.

[21] Tanuma M, Tanaka M, Orii T (2016). Contribution of ward pharmacists to appropriate antimicrobial use in the hematology ward. Jpn J Chemother..

[22] Costelloe C, Metcalfe C, Lovering A, Mant D, Hay AD (2010). Effect of antibiotic prescribing in primary care on antimicrobial resistance in individual patients: systematic review and meta-analysis. BMJ.

[23] Bell BG, Schellevis F, Stobberingh E, Goossens H, Pringle M (2014). A systematic review and meta-analysis of the effects of antibiotic consumption on antibiotic resistance. BMC Infect Dis.

[24] Matsuda S (2016). Development of Case Mix Based Evaluation System in Japan. Jpn Hosp..

[25] Japan Nosocomial Infections Surveillance. JANIS Open Report 2016 (All Facilities). https://janis.mhlw.go.jp/english/report/open_report/2016/3/1/ken_Open_Report_Eng_201600_clsi2012.pdf. Accessed 27 July 2019.

[26] Quan H, Li B, Couris CM, Fushimi K, Graham P, Hider P, Januel JM, Sundararajan V (2011). Updating and validating the Charlson comorbidity index and score for risk adjustment in hospital discharge abstracts using data from 6 countries. Am J Epidemiol.

[27] AMR Clinical Reference Center. Antimicoribal agents’ master. http://amrcrc.ncgm.go.jp/surveillance/030/20190116rule.pdf. Accessed 27 July 2019.

[28] Lang A, De Fina G, Meyer R, Aschbacher R, Rizza F, Mayr O, Casini M (2001). Comparison of antimicrobial use and resistance of bacterial isolates in a haematology ward and an intensive care unit. Eur J Clin Microbiol Infect Dis.

[29] Rajmokan M, Morton A, Marquess J, Playford EG, Jones M (2013). Development of a risk-adjustment model for antimicrobial utilization data in 21 public hospitals in Queensland, Australia (2006–11). J Antimicrob Chemother.

[30] Muraki Y, Yagi T, Tsuji Y, Nishimura N, Tanabe M, Niwa T, Watanabe T, Fujimoto S, Takayama K, Murakami N (2016). Japanese antimicrobial consumption surveillance: first report on oral and parenteral antimicrobial consumption in Japan (2009–-2013). J Glob Antimicrob Resist..

[31] Chong Y, Shimoda S, Yakushiji H, Ito Y, Miyamoto T, Kamimura T, Shimono N, Akashi K (2013). Antibiotic rotation for febrile neutropenic patients with hematological malignancies: clinical significance of antibiotic heterogeneity. PLoS ONE.

[32] Mikulska M, Averbuch D, Tissot F, Cordonnier C, Akova M, Calandra T, Ceppi M, Bruzzi P, Viscoli C, Aljurf M, Averbuch D (2018). Fluoroquinolone prophylaxis in haematological cancer patients with neutropenia: ECIL critical appraisal of previous guidelines. J Infect.

[33] Kimura SI, Fujita H, Kato H, Hiramoto N, Hosono N, Takahashi T, Shigeno K, Hatsumi N, Minamiguchi H, Miyatake J (2017). Management of infection during chemotherapy for acute leukemia in Japan: a nationwide questionnaire-based survey by the Japan Adult Leukemia Study Group. Support Care Cancer.

[34] Hicks LA, Bartoces MG, Roberts RM, Suda KJ, Hunkler RJ, Taylor TH, Schrag SJ (2015). US outpatient antibiotic prescribing variation according to geography, patient population, and provider specialty in 2011. Clin Infect Dis.

[35] Pien BC, Sundaram P, Raoof N, Costa SF, Mirrett S, Woods CW, Reller LB, Weinstein MP (2010). The clinical and prognostic importance of positive blood cultures in adults. Am J Med.

[36] Mimura W, Fukuda H, Konomura K, Akazawa M. Assessment of record linkage using administrative claims data and antimicrobial susceptibility testing data. ISPE’s 12 the Asian Conference on Pharmacoepidemiology and 25th Japanese Conference on Pharmacoepidemiology joint meeting. 2019. Kyoto, Japan. http://www.jtbw-mice.com/acpe2019/data/pdf/poster_abstracts_ACPE2019.pdf. Accessed 26 Dec 2019.

