# Supplementary material for: Antimicrobial utilization and antimicrobial resistance in patients with haematological malignancies in Japan: a multi-centre cross-sectional study
Source: Ann Clin Microbiol Antimicrob. 2020 Feb 17;19:7. doi: 10.1186/s12941-020-00348-0 (PMC7027235; doi:10.1186/s12941-020-00348-0)
Supplement: Supplementary file 1 — Additional file 1: Table S1. Definitions of variables. [file 12941_2020_348_MOESM1_ESM.docx]

## **Table S1. Definitions of variables**

| Variable | Definition |
| --- | --- |
| Central venous catheter | 130004410, central venous injection; 130004410, central venous injection; 130004670, insertion of central venous catheters; 130007510, continuous local venous infusion of anti-cancer; 130008510, central venous injection (central venous access port); 130009070, central venous access port placement (limb); 130009170, central venous access port placement (head and neck); 130010150, port placement of continuous local venous infusion of anti-cancer (limb); 130010250, port placement of continuous local venous infusion of anti-cancer (head and neck); or 130011610, insertion of peripherally inserted central catheter |
| Urinary catheter | 140013110, bladder irrigation (lavage); 140013350, irrigation of other indwelling urinary catheter; 140013810, insertion of indwelling urinary catheter; or 130013950, replacement of indwelling urinary catheter |
| Cleanroom | 190106570 and 190146510 |
| Hematopoietic stem cell transplantation | allogeneic (150225910, autologous bone marrow transplant; 150297810, allogeneic hematopoietic stem cell transplant; or 150349810, umbilical cord blood transplant) and autologous (150266410, autologous hematopoietic stem cell transplant or 150266310, autograft bone marrow transplant) |
