# Supplementary material for: Antimicrobial utilization and antimicrobial resistance in patients with haematological malignancies in Japan: a multi-centre cross-sectional study
Source: Ann Clin Microbiol Antimicrob. 2020 Feb 17;19:7. doi: 10.1186/s12941-020-00348-0 (PMC7027235; doi:10.1186/s12941-020-00348-0)
Supplement: Supplementary file 2 — Additional file 2: Table S2. Definitions of antibiotic-resistant bacteria. [file 12941_2020_348_MOESM2_ESM.docx]

## **Table S2. Definitions of antibiotic-resistant bacteria**

| Isolated Bacterium | Definition | broth microdilution method |
| --- | --- | --- |
| Multidrug-resistant *P. aeruginosa* (MDRP) | *P. aeruginosa* satisfying all of the following criteria:  1. Resistant to carbapenems (IPM or MEPM)  2. Resistant to aminoglycosides (AMK)  3. Resistant to fluoroquinolones (NFLX, OFLX, LVFX, CPFX, or LFLX) | 1. IPM ≥16μg/mL or MEPM ≥16μg/mL  2. AMK ≥32μg/mL  3. NFLX ≥16μg/mL, OFLX ≥8μg/mL, LVFX ≥8μg/mL, LFLX ≥8μg/mL, or CPFX ≥4μg/mL |
| Multidrug-resistant *Acinetobacter* spp. (MDRA) | *Acinetobacter* spp. satisfying all of the following criteria:  1. Resistant to carbapenems (IPM or MEPM)  2. Resistant to aminoglycosides (AMK)  3. Resistant to fluoroquinolones (LVFX or CPFX) | 1. IPM ≥16μg/mL, MEPM ≥16μg/mL  2. AMK ≥32μg/mL†  3. LVFX ≥8μg/mL or CPFX ≥4μg /mL |
| Carbapenem-resistant Enterobacteriaceae (CRE) | Enterobacteriaceae satisfying one of the following criteria.  1. Resistant to MEPM  2. Resistant to IPM and also resistant to CMZ | 1. MEPM ≥2μg/mL  2. IPM ≥2μg/mL and CMZ ≥64μg/mL |
| Carbapenem-resistant *P. aeruginosa* | *P. aeruginosa* resistant to IPM or MEPM | IPM ≥16μg /mL or MEPM ≥16μg /mL |
| Third-generation cephalosporin-resistant *K. pneumoniae* | *K. pneumoniae* resistant to CTX, CTRX, or CAZ | CTX ≥4μg /mL, CTRX ≥4μg /mL, or CAZ ≥16μg/mL |
| Third-generation cephalosporin-resistant *E. coli* | *E. coli* resistant to CTX, CTRX, or CAZ | CTX ≥4μg /mL, CTRX ≥4μg /mL, or CAZ ≥16μg/mL |
| Fluoroquinolone-resistant *E. coli* | *E. coli* resistant to fluoroquinolones (NFLX, OFLX, LVFX, LFLX, or CPFX) | NFLX ≥16μg /mL, OFLX ≥8μg /mL, LVFX ≥8μg /mL,  LFLX ≥8μg /mL, or CPFX ≥4μg /mL |
| Methicillin-resistant *Staphylococcus aureus* | MRSA or *S. aureus* satisfying all of the following criteria:  1. Resistant to MPIPC or CFX | MPIPC ≥4μg /mL or CFX ≥8μg /mL |

AMK, amikacin; CAZ, ceftazidime; CFX, cefoxitin; CMZ, cefmetazole; CPFX, ciprofloxacin; CTRX, ceftriaxone; CTX, cefotaxime; IPM, imipenem; LVFX, levofloxacin; MEPM, meropenem; MPIPC, oxacillin; NFLX, norfloxacin; OFLX, ofloxacin
