# Supplementary material for: Antimicrobial utilization and antimicrobial resistance in patients with haematological malignancies in Japan: a multi-centre cross-sectional study
Source: Ann Clin Microbiol Antimicrob. 2020 Feb 17;19:7. doi: 10.1186/s12941-020-00348-0 (PMC7027235; doi:10.1186/s12941-020-00348-0)
Supplement: Supplementary file 3 — Additional file 3: Table S3. Characteristics of patients with haematological malignancies stratified by the underlying disease. [file 12941_2020_348_MOESM3_ESM.docx]

# **Table S3. Characteristics of patients with haematological malignancies stratified by the underlying disease**

| Variables | HL | | NHL | | MM | | LL | | ML | | MDS | |
| --- | --- | --- | --- | --- | --- | --- | --- | --- | --- | --- | --- | --- |
|  | (n = 199) | | (n = 4028) | | (n = 1153) | | (n = 551) | | (n = 1224) | | (n = 909) | |
| Sex, n (%) |  |  |  |  |  |  |  |  |  |  |  |  |
| Male | 132 | (66.3) | 2201 | (54.6) | 596 | (51.7) | 290 | (52.6) | 760 | (62.1) | 599 | (65.9) |
| Female | 67 | (33.7) | 1827 | (45.4) | 557 | (48.3) | 261 | (47.4) | 464 | (37.9) | 310 | (34.1) |
| Age, years, median (Q1–Q3) | 63.0 | (45.0–74.0) | 70 | (62.0–78.0) | 71.0 | (64.0–78.0) | 63.0 | (49.0–73.0) | 66.0 | (53.0–75.0) | 76.0 | (67.0–82.0) |
| ≤17 years | 2 | (1.0) | 5 | (0.1) | 0 | (0.0) | 6 | (1.1) | 3 | (0.2) | 1 | (0.1) |
| 19–64 years | 106 | (53.3) | 1213 | (30.1) | 322 | (27.9) | 285 | (51.7) | 543 | (44.4) | 155 | (17.0) |
| 65–74 years | 48 | (24.1) | 1319 | (32.7) | 399 | (34.6) | 138 | (25.0) | 342 | (27.9) | 258 | (28.4) |
| ≥75 years | 43 | (21.6) | 1491 | (37.0) | 432 | (37.5) | 122 | (22.1) | 336 | (27.5) | 495 | (54.5) |
| CCI, n (%) |  |  |  |  |  |  |  |  |  |  |  |  |
| ≤2 | 157 | (78.9) | 3078 | (76.4) | 858 | (74.4) | 436 | (79.1) | 1005 | (82.1) | 860 | (94.6) |
| 3-5 | 37 | (18.6) | 779 | (19.3) | 253 | (21.9) | 83 | (15.1) | 198 | (16.2) | 42 | (4.6) |
| >5 | 5 | (2.5) | 171 | (4.2) | 42 | (3.6) | 32 | (5.8) | 21 | (1.7) | 7 | (0.8) |
| Follow-up duration. Days. median (Q1–Q3) | 59.0 | (20.0–194.0) | 97.0 | 26.0–209.0 | 87.0 | 27.0–267.0 | 134.0 | (39.0–268.0) | 101.0 | (25.0–223.0) | 85 | (24.0–235.0) |
| Total Length of stay, days, median (Q1-Q3) | 33.0 | (19.0–77.0) | 46.0 | 22.0–100 | 47.0 | 24.0–86.0 | 77.0 | (33.0–160.0) | 72.0 | (21.0–140.0) | 42 | (19.0–91.0) |
| Length of stay, days, median (Q1-Q3) | 16.0 | (7.0–26.0) | 17.0 | 10.0–25.0 | 22.0 | 12.0–33.0 | 25.0 | (13.0–41.0) | 30.0, | (15.0–43.0) | 13.0 | (9.0–27.0) |
| Chemotherapy, n (%) | 180 | (90.5) | 3592 | (89.2) | 960 | (83.3) | 452 | (82.0) | 941 | (76.9) | 417 | (45.8) |
| Febrile neutropenia, n (%) | 16 | (8.0) | 514 | (12.8) | 73 | (6.3) | 88 | (16.0) | 258 | (21.1) | 151 | (16.6) |
| G-CSF, n (%) | 97 | (48.7) | 2577 | (64.0) | 369 | (32.0) | 360 | (65.3) | 425 | (34.7) | 211 | (23.2) |
| Central venous catheter, n (%) | 76 | (38.2) | 1368 | (34.0) | 364 | (31.6) | 297 | (53.9) | 696 | (56.9) | 271 | (29.8) |
| Urinary catheter, n (%) | 31 | (15.6) | 873 | (21.7) | 365 | (31.7) | 191 | (34.7) | 380 | (31.0) | 269 | (29.6) |
| HSCT |  |  |  |  |  |  |  |  |  |  |  |  |
| Allogeneic, n (%) | 2 | (1.0) | 45 | (1.1) | 2 | (0.2) | 112 | (20.3) | 200 | (16.3) | 46 | (5.1) |
| Autologous, n (%) | 17 | (8.5) | 144 | (3.6) | 135 | (11.7) | 0 | (0.0) | 5 | (0.4) | 0 | (0.0) |
| Isolation room, n (%) | 37 | (18.6) | 860 | (21.4) | 233 | (20.2) | 268 | (48.6) | 703 | (57.4) | 251 | (27.6) |
| In-hospital mortality, n (%) | 23 | (11.6) | 635 | (15.8) | 222 | (19.3) | 159 | (28.9) | 403 | (32.9) | 319 | (35.1) |

HL, Hodgkin lymphoma; NHL, non-Hodgkin lymphoma; MM, Multiple myeloma; LL, Lymphoid leukaemia; ML, Myeloid leukaemia; MDS, Myelodysplastic syndromes; CCI, Charlson comorbidity index; LOS, length of stay; Granulocyte-colony stimulating factor; HSCT, hematopoietic stem cell transportation.
