# Supplementary material for: Antimicrobial utilization and antimicrobial resistance in patients with haematological malignancies in Japan: a multi-centre cross-sectional study
Source: Ann Clin Microbiol Antimicrob. 2020 Feb 17;19:7. doi: 10.1186/s12941-020-00348-0 (PMC7027235; doi:10.1186/s12941-020-00348-0)
Supplement: Supplementary file 4 — Additional file 4: Table S4. Antimicrobial use among underlying diseases [file 12941_2020_348_MOESM4_ESM.docx]

## **Table S4. Antimicrobial use among underlying diseases.**

|  | Total | HL | NHL | MM | LL | ML | MDS |
| --- | --- | --- | --- | --- | --- | --- | --- |
| Cefepime | 156.7 | 75.3 | 113.6 | 97.0 | 217.3 | 266.9 | 181.0 |
| Carbapenems | 104.8 | 52.7 | 54.9 | 48.1 | 150.9 | 209.2 | 175.3 |
| Glycopeptides | 48.0 | 30.2 | 23.2 | 19.4 | 72.8 | 104.9 | 70.9 |
| Piperacillin/Tazobactam | 28.4 | 31.4 | 18.6 | 21.0 | 32.3 | 45.8 | 46.0 |
| Third-generation cephalosporins | 16.8 | 12.2 | 14.7 | 23.0 | 11.5 | 15.5 | 27.4 |
| Quinolones | 8.8 | 4.3 | 5.3 | 5.2 | 10.1 | 14.6 | 17.9 |
| Penicillins | 7.5 | 4.3 | 6.3 | 10.4 | 9.1 | 5.8 | 11.7 |
| First/Second-generation cephalosporins | 4.0 | 6.0 | 4.4 | 4.3 | 3.4 | 2.6 | 4.5 |
| Others | 22.3 | 19.7 | 12.8 | 12.5 | 25.3 | 42.2 | 38.3 |
| Oral levofloxacin | 101.5 | 69.1 | 78.8 | 64.7 | 151.5 | 154.4 | 110.9 |

HL, Hodgkin lymphoma; NHL, non-Hodgkin lymphoma; MM, Multiple myeloma; LL, Lymphoid leukaemia; ML, Myeloid leukaemia; MDS, Myelodysplastic syndromes.
