# Supplementary material for: Antimicrobial utilization and antimicrobial resistance in patients with haematological malignancies in Japan: a multi-centre cross-sectional study
Source: Ann Clin Microbiol Antimicrob. 2020 Feb 17;19:7. doi: 10.1186/s12941-020-00348-0 (PMC7027235; doi:10.1186/s12941-020-00348-0)
Supplement: Supplementary file 5 — Additional file 5: Table S5. Infections caused by gram-negative bacteria stratified by the underlying disease. [file 12941_2020_348_MOESM5_ESM.docx]

## **Table S5. Infections caused by gram-negative bacteria stratified by the underlying disease**

|  | HL (n= 199) | | | | NHL (n= 4024) | | | | MM (n= 1153) | | | |
| --- | --- | --- | --- | --- | --- | --- | --- | --- | --- | --- | --- | --- |
|  | Any^a^  (n= 98) | | Blood  (n= 85) | | Any^a^  (n= 2059) | | Blood  (n= 1771) | | Any^a^  (n= 696) | | Blood  (n= 604) | |
| *E. coli* | 14 | (14.3) | 0 | (0.0) | 284 | (13.8) | 68 | (3.8) | 135 | (19.4) | 23 | (3.8) |
| *P. aeruginosa* | 3 | (3.1) | 0 | (0.0) | 114 | (5.5) | 30 | (1.7) | 45 | (6.5) | 13 | (2.2) |
| *Klebsiella* spp. | 11 | (11.2) | 2 | (2.4) | 170 | (8.3) | 24 | (1.4) | 79 | (11.4) | 8 | (1.3) |
| *Enterobacter* spp. | 5 | (5.1) | 1 | (1.2) | 93 | (4.5) | 15 | (0.8) | 34 | (4.9) | 6 | (1.0) |
| *Citrobacter* spp. | 2 | (2.0) | 0 | (0.0) | 70 | (3.4) | 2 | (0.1) | 25 | (3.6) | 0 | (0.0) |
| *Serratia marcescens* | 1 | (1.0) | 1 | (1.2) | 15 | (0.7) | 5 | (0.3) | 3 | (0.4) | 0 | (0.0) |
| *Proteus* spp. | 2 | (2.0) | 0 | (0.0) | 33 | (1.6) | 2 | (0.1) | 7 | (1.0) | 3 | (0.5) |
| *Acinetobacter* spp. | 3 | (3.1) | 0 | (0.0) | 35 | (1.7) | 9 | (0.5) | 15 | (2.2) | 1 | (0.2) |
|  | LL (n= 553) | | | | ML (n= 1234) | | | | MDS (n= 909) | | | |
|  | Any^a^  (n= 414) | | Blood  (n= 396) | | Any^a^  (n= 940) | | Blood  (n= 902) | | Any^a^  (n= 673) | | Blood  (n= 630) | |
| *E. coli* | 85 | (20.5) | 30 | (7.6) | 146 | (15.5) | 48 | (5.3) | 90 | (13.4) | 23 | (3.7) |
| *P. aeruginosa* | 27 | (6.5) | 5 | (1.3) | 45 | (4.8) | 15 | (1.7) | 48 | (7.1) | 10 | (1.6) |
| *Klebsiella* spp. | 52 | (12.6) | 17 | (4.3) | 86 | (9.1) | 34 | (3.8) | 57 | (8.5) | 12 | (1.9) |
| *Enterobacter* spp. | 19 | (4.6) | 7 | (1.8) | 44 | (4.7) | 11 | (1.2) | 24 | (3.6) | 8 | (1.3) |
| *Citrobacter* spp. | 25 | (6.0) | 4 | (1.0) | 29 | (3.1) | 2 | (0.2) | 16 | (2.4) | 1 | (0.2) |
| *Serratia marcescens* | 1 | (0.2) | 0 | (0.0) | 10 | (0.8) | 3 | (0.2) | 8 | (0.9) | 0 | (0.0) |
| *Proteus* spp. | 5 | (1.4) | 0 | (0.0) | 8 | (0.9) | 0 | (0.0) | 3 | (0.4) | 0 | (0.0) |
| *Acinetobacter* spp. | 6 | (1.4) | 2 | (0.5) | 22 | (2.3) | 7 | (0.8) | 9 | (1.3) | 1 | (0.2) |

HL, Hodgkin lymphoma; NHL, non-Hodgkin lymphoma; MM, Multiple myeloma; LL, Lymphoid leukaemia; ML, Myeloid leukaemia; MDS, Myelodysplastic syndromes.

^a^Any include all type of specimens (blood, respiratory, urine, stool, cerebrospinal fluid, and others).
