# Supplementary material for: Antimicrobial utilization and antimicrobial resistance in patients with haematological malignancies in Japan: a multi-centre cross-sectional study
Source: Ann Clin Microbiol Antimicrob. 2020 Feb 17;19:7. doi: 10.1186/s12941-020-00348-0 (PMC7027235; doi:10.1186/s12941-020-00348-0)
Supplement: Supplementary file 6 — Additional file 6: Table S6. Infections caused by antibiotic-resistant bacteria stratified by the underlying disease. [file 12941_2020_348_MOESM6_ESM.docx]

**Table S6. Infections caused by antibiotic-resistant bacteria stratified by the underlying disease**

|  | HL (n= 199) | | | | NHL (n= 4028) | | | | MM (n= 1153) | | | |
| --- | --- | --- | --- | --- | --- | --- | --- | --- | --- | --- | --- | --- |
|  | Any^a^  (n= 98) | | Blood  (n= 85) | | Any^a^  (n= 2062) | | Blood  (n= 1772) | | Any^a^  (n= 698) | | Blood  (n= 605) | |
| Multidrug-resistant *P. aeruginosa* | 0 | (0.0) | 0 | (0.0) | 1 | (0.0) | 0 | (0.0) | 1 | (0.1) | 0 | (0.0) |
| Multidrug-resistant *Acinetobacter spp.* | 0 | (0.0) | 0 | (0.0) | 0 | (0.0) | 0 | (0.0) | 0 | (0.0) | 0 | (0.0) |
| Carbapenem-resistant Enterobacteriaceae | 0 | (0.0) | 0 | (0.0) | 9 | (0.2) | 0 | (0.0) | 2 | (0.2) | 1 | (0.1) |
| Carbapenem-resistant *P. aeruginosa* | 2 | (1.0) | 0 | (0.0) | 14 | (0.3) | 3 | (0.1) | 6 | (0.5) | 2 | (0.2) |
| Third-generation cephalosporin-resistant *K. pneumoniae* | 1 | (0.5) | 0 | (0.0) | 20 | (0.5) | 1 | (0.0) | 8 | (0.7) | 4 | (0.3) |
| Third-generation cephalosporin-resistant *E. coli* | 3 | (1.5) | 0 | (0.0) | 63 | (1.6) | 18 | (0.4) | 27 | (2.3) | 2 | (0.2) |
| Fluoroquinolone-resistant *E. coli* | 5 | (2.5) | 0 | (0.0) | 103 | (2.6) | 28 | (0.7) | 43 | (3.7) | 6 | (0.5) |
| Methicillin-resistant *S. aureus* | 5 | (2.5) | 3 | (1.5) | 72 | (1.8) | 11 | (0.3) | 35 | (3.0) | 5 | (0.4) |
|  | LL (n= 551) | | | | ML (n= 1224) | | | | MDS (n= 909) | | | |
|  | Any^a^  (n= 415) | | Blood  (n= 397) | | Any^a^  (n= 940) | | Blood  (n= 902) | | Any^a^  (n= 673) | | Blood  (n= 630) | |
| Multidrug-resistant *P. aeruginosa* | 0 | (0.0) | 0 | (0.0) | 0 | (0.0) | 0 | (0.0) | 0 | (0.0) | 0 | (0.0) |
| Multidrug-resistant *Acinetobacter spp.* | 0 | (0.0) | 0 | (0.0) | 0 | (0.0) | 0 | (0.0) | 0 | (0.0) | 0 | (0.0) |
| Carbapenem-resistant Enterobacteriaceae | 0 | (0.0) | 0 | (0.0) | 2 | (0.2) | 2 | (0.2) | 2 | (0.2) | 0 | (0.0) |
| Carbapenem-resistant *P. aeruginosa* | 6 | (1.1) | 2 | (0.4) | 14 | (1.1) | 5 | (0.4) | 3 | (0.3) | 1 | (0.1) |
| Third-generation cephalosporin-resistant *K. pneumoniae* | 3 | (0.5) | 0 | (0.0) | 6 | (0.5) | 4 | (0.3) | 4 | (0.4) | 1 | (0.1) |
| Third-generation cephalosporin-resistant *E. coli* | 23 | (4.2) | 14 | (2.5) | 32 | (2.6) | 12 | (1.0) | 19 | (2.1) | 5 | (0.6) |
| Fluoroquinolone-resistant *E. coli* | 43 | (7.8) | 27 | (4.9) | 55 | (4.5) | 27 | (2.2) | 42 | (4.6) | 16 | (1.8) |
| Methicillin-resistant *S. aureus* | 17 | (3.1) | 9 | (1.6) | 39 | (3.2) | 10 | (0.8) | 42 | (4.6) | 7 | (0.8) |

HL, Hodgkin lymphoma; NHL, non-Hodgkin lymphoma; MM, multiple myeloma; LL, lymphoid leukaemia; ML, myeloid leukaemia; MDS, myelodysplastic syndromes. ^a^Any include all type of specimens (blood, respiratory, urine, stool, cerebrospinal fluid, and others)
